# Iron Metabolism in Pancreatic Beta-Cell Function and Dysfunction

**DOI:** 10.3390/cells10112841

**Published:** 2021-10-22

**Authors:** Algerta Marku, Alessandra Galli, Paola Marciani, Nevia Dule, Carla Perego, Michela Castagna

**Affiliations:** Department of Excellence Pharmacological and Biomolecular Sciences, Università degli Studi di Milano, Via Trentacoste, 22134 Milano, Italy; algerta.marku@unimi.it (A.M.); alessandra.galli1@unimi.it (A.G.); paola.marciani@unimi.it (P.M.); nevia.dule@unimi.it (N.D.)

**Keywords:** Iron metabolism, beta-cell function, reactive oxygen species, diabetes

## Abstract

Iron is an essential element involved in a variety of physiological functions. In the pancreatic beta-cells, being part of Fe-S cluster proteins, it is necessary for the correct insulin synthesis and processing. In the mitochondria, as a component of the respiratory chain, it allows the production of ATP and reactive oxygen species (ROS) that trigger beta-cell depolarization and potentiate the calcium-dependent insulin release. Iron cellular content must be finely tuned to ensure the normal supply but also to prevent overloading. Indeed, due to the high reactivity with oxygen and the formation of free radicals, iron excess may cause oxidative damage of cells that are extremely vulnerable to this condition because the normal elevated ROS production and the paucity in antioxidant enzyme activities. The aim of the present review is to provide insights into the mechanisms responsible for iron homeostasis in beta-cells, describing how alteration of these processes has been related to beta-cell damage and failure. Defects in iron-storing or -chaperoning proteins have been detected in diabetic conditions; therefore, the control of iron metabolism in these cells deserves further investigation as a promising target for the development of new disease treatments.

## 1. Introduction

Iron plays a central role in a variety of essential cellular functions as oxygen transport and exchange, being the metal component of many intracellular enzymes. Its ability to react with oxygen also makes it a toxic compound, able to generate reactive oxygen species (ROS) that can damage DNA, phospholipids and proteins. It is therefore of utmost importance, for both the cells and the organisms, to maintain iron homeostasis, ensuring iron supply and preventing accumulation of iron excess. As a matter of fact, several disease states are characterised by aberrant iron handling. Abnormal iron homeostasis has been detected in hemochromatosis, anaemia, atherosclerosis and in neurological diseases, such as Parkinson’s, Alzheimer’s, Huntington’s, Friedreich’s ataxia and the eating disorder pica [1,2,3,4,5,6,7,8,9,10,11].

Increasing evidence also points to a causal role of iron in diabetes. Iron is essential for insulin secretion [12,13], yet its accumulation is an important determinant of pancreatic islet inflammation and is considered a biomarker of diabetes risk and mortality [14].

The link between iron and diabetes first emerged considering pathological conditions as hemochromatosis and beta thalassemia [15,16,17,18], in which an involvement of iron overload in both beta-cell failure and insulin resistance was highlighted.

In addition, in type 2 diabetes mellitus (T2DM) subjects, increased levels of ferritin [19], a biomarker of increased body iron stores, and reduced levels of hepcidin, the hepatic hormone responsible of the systemic iron homeostasis, have been detected in the blood, highlighting the systemic alteration of iron metabolism [20].

Despite the accruing evidence, the molecular mechanisms linking iron excess condition and diabetes is not yet completely understood. While its homeostasis may impact different organs, the islets of Langerhans seem particularly vulnerable to iron. ROS accumulation occurring in the presence of excess iron can induce oxidative damage of pancreatic beta-cells. The strong susceptibility to oxidative stress of these cells is a consequence of their high metabolic activity [21,22], and high rate of ROS production, coupled with their weak defence mechanisms against oxidative insults. Beta-cells indeed are characterised by a reduced expression of superoxide dismutase, catalase and glutathione peroxidase [21,23]. Given the increasing beta-cell failure in diabetes development and progression, in the present review, we describe recent advancements in the comprehension of iron handling in these cells and the role of iron and iron-interacting proteins in beta-cell physiology and pathology. A better understanding of iron metabolism and homeostasis in these cells may be helpful in the development of new therapies to treat diabetes mellitus.

## 2. Iron-Homeostasis in Beta-Cells

**Figure 1:**
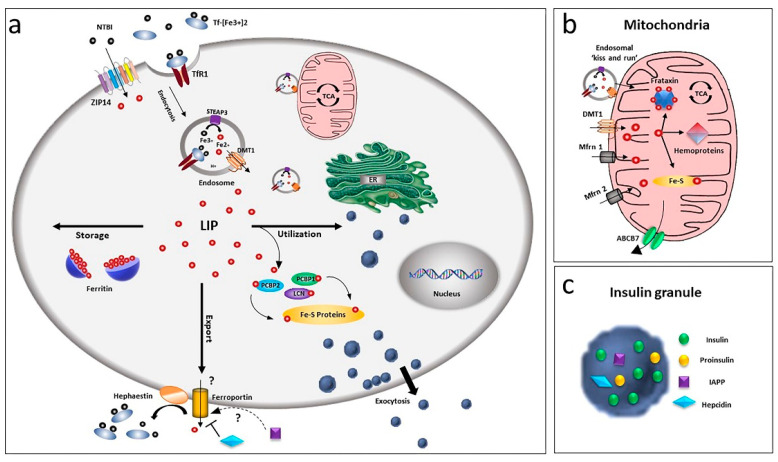
Overview of iron homeostasis in beta-cells. (**a**) Iron uptake in beta-cells is mediated by endocytosis of the transferrin-transferrin receptor complex and its release from endosomes by the divalent metal ion transporter DMT1. As non-transferrin-bound iron (NTBI), it can also be imported by the zinc transporter ZIP14 transporter. Being toxic as a free ion, Fe^2+^ is then readily distributed for storage, bound to ferritin or for utilisation by chaperoning proteins as PCBPs and lipocalin. Iron efflux is mediated by ferroportin, a process regulated by hepcidin and hephaestin. LIP: labile iron pool. (**b**) Within the cell, the major site of utilisation is the mitochondria, where the ion is transported via DMT1 and mitoferrin (Mfrn1, Mfrn2) and inserted into heme and Fe/S cluster prosthetic groups. Mitochondria iron efflux is probably mediated by the ATP-binding cassette (ABC) transporter ABCB7. (**c**) Beta-cells, together with insulin, release IAPP and hepcidin, involved in a possible modulation of iron metabolism by an autocrine mechanism, via regulation of ferroportin.

Due to its chemical nature and its possible harmful effects, cells have developed a complex system to handle iron: carriers and receptors bind and transport the ion across the membranes, enzymes and buffering proteins control its redox state and free level and iron regulatory proteins modulate the expression of iron-binding proteins, according to the ion level. Pancreatic beta-cells possess several of these proteins, although some specific players of this relevant process are not yet completely defined (Figure 1). 

### 2.1. Iron Influx through the Plasma Membrane

Uptake of iron in beta-cells is performed by two different systems: a receptor-mediated transport for the transferrin-bound iron (TBI) and a non-transferrin-bound iron (NTBI) transport. The first mechanism is based on the interaction of transferrin-bound iron with the specific cell surface transferrin receptor 1 (TfR1) [13,24]. The complex is then internalised in endocytic compartments in conjunction with the divalent metal ion transporter DMT1 (or SLC11A2) and the metalloreductase six transmembrane epithelial antigen of the prostate family member 3 (STEAP3) [25].

Internalised vesicles then fuse with lysosomal compartments, and the acidic milieu prompts the conformational change of Tf-Fe complex and the release of Fe^3+^, enabling its reduction to the ferrous form by STEAP3. Fe^2+^ is extruded in the cytoplasm through DMT1, exploiting the H^+^ gradient created by the vacuolar H^+^-ATPase (v-ATPase) as the driving force [26]. 

Recently, a non-transferrin-bound iron (NTBI) uptake has also been described in the human beta-cell line βlox5 [27]. The chemical nature of plasma NTBI is not known but is believed to mainly exist in ferric citrate and other low-molecular-weight species [28,29]. In some pathological conditions, higher molecular weight NTBI plasma fractions have been detected, suggesting the possible binding of Fe^2+^ and Fe^3+^ to proteins [29] and the existence of different NTBI pools, depending on the iron overload conditions [30]. NTBI can be observed in the blood of patients with iron overload conditions when transferrin is saturated [31], although its presence has also been detected at not fully saturated transferrin levels [32]. Interestingly, in diabetic subjects NTBI is already present at transferrin saturations below 60% [33]. In primary human islets, NTBI uptake is mediated by the zinc transporter ZIP14 (SLC39A14), which localises to the plasma membrane of beta-cells, where iron loading is restricted. Chronic (24 h) high glucose levels upregulate the transporter expression, thus confirming the functional relevance of ZIP14 and suggesting possible consequences in iron homeostasis [34]. However, siRNA-mediated ZIP14 knockdown determined only a 50% reduction of NTBI uptake, suggesting that other transport systems may be involved as well. A role of L-type or T-type calcium channels seems unlikely due to the lack of iron overload in murine beta-cells expressing them [35].

### 2.2. Iron Efflux through the Plasma Membrane

The exit of iron from beta-cells is controversial: ferroportin/Ireg1 (FPN1, SLC40A1) so far is the only known exporter for iron [36,37], and islets show a very low immunoreactivity for this transporter [38], although they express hephaestin. This protein is responsible for the membrane stabilisation of ferroportin and the oxidation of Fe^2+^ to Fe^3+^ required for the interaction with transferrin [38]. 

Interestingly, beta-cells, together with insulin, also release hepcidin that is known to bind ferroportin and induce its internalisation [39,40], thus suggesting a positive feedback mechanism in iron regulation during glucose-stimulated insulin secretion, mediated by ferroportin control [41]. 

Another possible modulator of ferroportin is the islet amyloid polypeptide (IAPP) [42], which is released together with insulin and plays a role in glucose homeostasis [43] and in the control of food intake [44,45]. Although its role in iron homeostasis in beta-cells has not yet been established, it could suggest a parallelism with neurons, in which the amyloid polypeptide APP stabilises ferroportin at the plasma membrane and stimulates iron release through ferroxidase activity [46,47,48,49,50,51], thus preventing iron overload and oxidative stress.

### 2.3. Iron Binding Proteins

By a tight control of iron homeostasis, cells avoid excess of harmful free iron. Once inside the cell, iron forming the cytoplasmic labile pool (LIP) is sequestered by ferritin, the exclusive cytosolic iron-storage protein. Both H and L chains are expressed in beta-cells and modulated at the translational level by iron overload: when iron increases, ferritin synthesis increases as well as iron storage [52]. By sequestering the element, ferritin play a role in iron detoxification and functions as an iron reserve protein. Although the presence of a cytoplasmic labile iron pool consisting of chelatable iron has been detected in the past, concerns have been raised that iron, once internalised in cells, is delivered to ferritin via direct protein-protein interactions in a hydrophobic microenvironment, since LIP does not seem to have the chemical characteristics of an intermediate iron pool [53]. Chaperone proteins, such as poly r(C)-binding proteins (PCBPs) [54], are involved in this process.

All four known PCBP isoforms can bind and deliver iron to the cytosolic ferritin [52,55], but they show different abilities as iron chaperones. For example, only PCBP2 can bind to the carrier systems DMT1 and FPN1 in an iron-dependent way [56,57]. Both PCBP1 and PCBP2 can deliver iron to ferritin, but only PCBP1 is fundamental in ferritinophagy, an iron recycling process [58] in which the iron-ferritin complex is captured by the nuclear receptor coactivator-4 (NCOA4) and directed into the autophagosome [59]. 

The expression of both PCBP1 and PCBP2 has been documented in beta-cells, but their specific role in iron handling and whether they are also involved in iron delivery to intracellular organelles and Fe-S proteins remains to be elucidated in this cell type.

### 2.4. Iron Exchange with Organelles 

Although iron has been detected in almost all intracellular organelles, mitochondria are the main station of cellular iron metabolism. They are indeed a site of iron storage and utilisation. Vital synthesis of heme and iron-sulphur (Fe-S) clusters for electron transport proteins take place within them.

The iron exchange with mitochondria is thought to be mediated by DMT1 and the classical mitochondrial iron transporters mitoferrin (Mfrn) 1 and 2 [60], being the second more specific for non-erythroid cells [61]. Lipocalin (LCN) protein 2 is also involved in this process as a chaperon protein [13,62]. In HEK293 cells, permanently expressing DMT1, the transporter is present at the outer mitochondrial membrane (OMM) [63] and found to be involved in Fe^2+^ and Mn^2+^ uptake [64]. Mfrn1 and 2 ensure the iron transport across the inner mitochondrial membrane, where the element is utilised for heme synthesis and Fe-S clusters biogenesis or is sequestered by mitochondrial ferritin (MTFT). 

Fe-S cluster biogenesis requires frataxin, an iron mitochondrial chaperone expressed in islets and beta-cells and stimulated by hyperglycaemic conditions [65]. Individuals affected by Friedreich’s ataxia (FRDA), a neurodegenerative disorder caused by frataxin deficiency, also develop non-neurological symptoms, such as diabetes or glucose intolerance (8 to 32% incidence) [66]. In these patients, iron overload and increased beta-cell apoptosis have been observed, thus further supporting a link between iron dyshomeostasis and diabetes. 

Considering the exit of iron from the mitochondrial matrix, the ATP-binding cassette (ABC) transporter ABCB7 is believed to export iron in the form of Fe-S clusters. This hypothesis is based on the activity of the yeast orthologue Atm1 [67] that can transport glutathione-coordinated Fe-S clusters, connecting the mitochondrial and cytosolic Fe-S cluster assembly systems [68,69]. Recently, Pearson et al. confirmed this substrate specificity, highlighting the role of Mg-ATP in the transport process [70]. An additional mechanism for the exit of iron from the mitochondrial matrix could be the export of heme by specific transporters [71]. 

Iron can also be delivered to mitochondria by direct communication with other organelles. In developing erythroid cells, requiring a very efficient delivery of iron to mitochondria for heme synthesis, a direct delivery of iron from endosomes to mitochondria by a “kiss and run” mechanism [72]) has also been described, in which the transfer of the cation would be mediated by the docking of mitochondria and transferrin-loaded endosomes through the voltage dependent anion channel 1 (VDAC1) or DMT1 [64,73]. Due to the relevant role played by iron in insulin release (see below), similar mechanisms of iron delivery could also be envisaged for beta-cells, considering that the same process has been described in epithelial cells [74].

Contact sites between mitochondria and lysosomes, not related to mitophagy or lysosomal degradation of mitochondrial vesicles, have also been described by high-resolution microscopy [75]. Supporting the functional relevance of such a contact in iron transport, in erythroid progenitors, where the TfR2 isoform mediates the delivery of lysosomal transferrin to mitochondria, TfR2 deficiency reduced mitochondrial size and heme production [76]. Furthermore, in fibroblasts of patients affected by neurodegeneration with brain iron accumulation, mitochondrial function abnormalities and reduced lysosomal proteolytic activity have been observed [77], suggesting a further mechanism of intracellular iron trafficking based on the interaction between mitochondria and lysosomes. 

Mitochondrial-associated ER membranes (MAMs) could be also implicated in cell iron homeostasis. Deficiency of Cisd2 (CDGSH iron sulphur domain 2), an Fe-S protein localised on MAMs, leads to mitochondrial dysfunction and disturbance of intracellular Ca^2+^ homeostasis, resulting in insulin insensitivity in adipocytes [78]. Interestingly, in yeast, loss of the protein complex ERMES (endoplasmic reticulum mitochondria encounter structure) connecting the two organelles, determines an iron-deficiency response even in iron-repleted conditions, causing iron excess in the cell [79]. Furthermore, dominant mutants of the vacuolar protein sorting 13 (VSP13p) rescue ERMES mutants, suppressing the iron deficiency response. No transporters for the delivery of iron to endoplasmic reticulum (ER) have been identified so far. The 2Fe-2S protein iron sulphur domain 2 (Miner 1) that localises to ER in other cell types and is relevant for ER integrity [80,81] could be involved in this function [82]. 

### 2.5. Iron Metabolism Regulatory Proteins

As both iron deficiency and overload can be detrimental, in beta-cells, iron-genes are post-transcriptionally regulated by the iron regulatory proteins (IRPs), based on iron availability [83,84,85]. These are RNA-binding proteins that, by binding to IRE sequences present on mRNAs of iron handling proteins, modulate their translation. In particular, in conditions of iron deficiency, IRP binds to TfR1, DMT1 and ferritin mRNAs and promotes their translation, thus increasing cellular iron absorption and iron storage [83]. At the same time, IRPs suppress FPN1 translation, thus reducing cellular iron release [86]. Both IRP1 and IRP2 are expressed in beta-cells, and IRP2 knockout mice develop diabetes due to misregulation of iron metabolism as discussed later on. [87]. 

## 3. Iron Is Required for the Normal Beta-Cell Function

**Figure 2:**
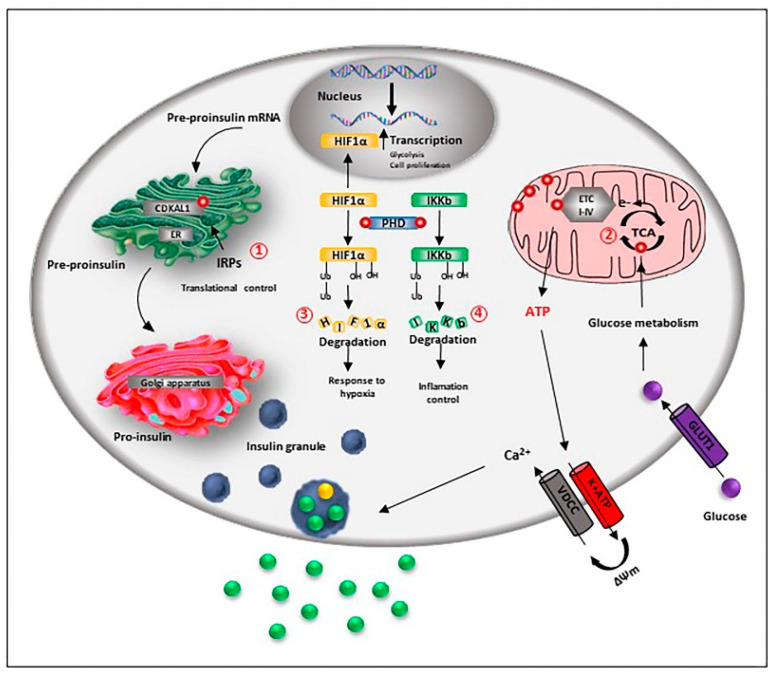
Iron is relevant for beta-cell function and preservation. Beta-cell iron homeostasis is under the control of the iron regulatory proteins (IRPs). Fe-S-cluster proteins are necessary for the correct processing and synthesis of insulin. The Fe-S cluster enzyme CDKAL1 is responsible for the adenosine methylthiolation in the tRNA for lysine, a modification required for the pro-insulin translational fidelity in pancreatic beta-cells. CDKAL1 is under the control of IRP2 (1). Iron is also essential for the metabolic coupling of insulin release: obligatory steps of the tricarboxylic acid (TCA) cycle are mediated by iron-dependent enzymes, and Fe-S cluster proteins are part of the respiratory chain complexes, allowing the synthesis of ATP (2). As a co-factor of the prolyl and asparaginyl hydroxylase (PHD), iron controls the degradation of hypoxia-inducible factor HIF-1α factor, influencing beta-cell response to hypoxia (3). Iron is also necessary to control inflammation, as PHD hydroxylates and inactivates the inhibitor of κB kinase (IKKb), an important upstream regulator of nuclear factor (NF)-kB (NF-kB) pathway, the major pro-inflammatory pathway in beta-cells (4).

Beta-cells express higher levels of iron import and storage proteins and show an increased iron metabolism compared to alpha- and delta-cells. This is because iron is a cofactor of several enzymes and an essential component of Fe-S cluster proteins involved in relevant functions ranging from insulin secretion to beta-cell proliferation and differentiation (Figure 2).

In line with this possibility, iron-depleted mouse islets show impaired glucose-stimulated insulin release and human beta-cells upregulate transferrin receptor surface expression in conditions of glucose depletion [88]. 

Insulin synthesis and secretion are exquisitely dependent on iron. Pro-insulin translational fidelity in pancreatic beta-cells requires the activity of the Fe-S cluster enzyme CDKAL1. This enzyme is responsible for the adenosine methylthiolation in the tRNA for lysine, a modification required to maintain the accuracy of codon recognition during protein translation. CDKAL1 dysfunction causes a misreading of the codon and impaired proinsulin processing and release [87]. Interestingly, mice lacking IRP2 protein develop diabetes because the consequent iron deficiency leads to a reduced function of CDKAL1 [87].

Iron is also required for the efficient coupling between glucose metabolism and insulin release. A key step in this mechanism is the glucose oxidation in the tricarboxylic acid (TCA) cycle to produce reducing equivalents, which are utilised by the respiratory chain to generate the proton gradient that drives the ATP synthesis. The resulting increase in the ATP/ADP ratio leads to ATP-dependent potassium channel closure and membrane depolarisation; the opening of voltage-gated calcium channels follows and promotes insulin secretion. Iron is involved in the TCA cycle since the succinate dehydrogenase and aconitase, which catalyse obligatory steps in the cycle, are both Fe-dependent enzymes. Iron also directly controls ATP synthesis because, as an Fe-S cluster protein, it is part of the complexes I, II, III and IV of the mitochondrial respiratory chain. In line with this possibility, in the Ins-1E-β-cell line, ZIP14 silencing decreases iron transport into the cells and downregulates the expression of many metal-binding proteins, such as the cytoplasmic iron-sensing protein aconitase 1 (ACO1) and ribosomal mitochondrial proteins, thus affecting oxidative phosphorylation and insulin release [34].

Iron can also indirectly modulate insulin release, through ROS generation. Several steps in the insulin release are sensitive to the redox balance; for example the plasma membrane depolarisation, triggered by the closure of ATP-dependent K^+^-channels, is supported by the activation of the redox-gated non-specific cation channel NSCC [89]. Furthermore, during the insulin granule fusion, the action of voltage-gated calcium channels is amplified by the calcium release from the ER, through a ROS-dependent activation of the ryanodine receptor 2 [22,90,91]. 

Iron also regulates other aspects of beta-cell physiology, such as the proliferation, differentiation, and survival. As a co-factor of the prolyl and asparaginyl hydroxylase (PHD), iron controls the hypoxia-inducible factor HIF-1α degradation and participates in the beta-cell response to low oxygen conditions [92,93]. Under normoxia, PHD hydroxylates HIF-1α, thus causing its degradation; under hypoxia or iron depletion, PHD is inactive, and HIF-1α shuttles to the nucleus and controls the transcription of a number of genes involved in the regulation of the glycolytic pathway. As a consequence, the cellular metabolism is modified, and cells shift from a proliferative to a resting state. Supporting this role, iron depletion due to lysosomal dysfunction, causes the activation of HIF-1α signalling and a consequent proliferation impairment [94]. 

Through its relationship with HIF-1α, iron could also participate in the regulation of beta-cell functions mediated by the circadian clock mechanism. The glucose metabolism and insulin release are under the control of this mechanism in beta-cells [95,96] and circadian disruption is involved in T2DM development in both rodents and humans [97,98,99,100]. Indeed, a reciprocal interaction between the clock genes and the HIF-1α transcriptional programs seems evident given that HIF-1α can bind the promoter region of clock genes and control their transcription, at least in muscle cells [101], and, conversely, HIF-1α is a direct transcriptional target of the orthologue of Clock gene, NPAS2, in hepatocellular carcinoma [102]. Furthermore, several iron-related genes and PHD itself are transcriptionally regulated by the clock genes [103,104], suggesting a circadian regulation of iron homeostasis, probably important to govern rhythmic tissue-specific metabolic reprogramming, based on oxygen and fuel availability. Interestingly, changes in Clock, NPAS2 and Baml1 expression have also been observed between newborn and adult rat islets, since the acquisition of a circadian control of insulin release allows immature islets, characterised by amino acid-stimulated insulin biosynthesis and release, to achieve the mature ability of secreting insulin in response to elevated glucose concentrations [105]. 

A correct iron intake is also necessary in beta-cells to control the inflammation, as the iron-dependent PHD hydroxylates and inactivates the inhibitor of κB kinase (IKKb), an important upstream regulator of the nuclear factor (NF)-kB (NF-kB) pathway, the major pro-inflammatory pathways in beta-cells [106,107].

Recent data suggest a possible involvement of iron in beta-cell differentiation. Indeed, TfR1 levels, transferrin-bound iron uptake and ferritin transcripts are upregulated in the early post-natal weeks of beta-cell maturation [88]. This observation indicates an increased requirement of iron exactly during the metabolic switch from aerobic glycolysis to oxidative phosphorylation, necessary for beta-cell maturation [108,109]. As reported above, several iron-dependent proteins are required to sustain the activity of the fully mature functional beta-cell.

Recently, our laboratory has described how biophysical characteristics of the extracellular environment can also influence cell differentiation and survival of human islets and beta-cells [110,111]. We found that the extracellular matrix nanotopography, via a mechanotransduction pathway which involves mechanosensitive integrins, reorganisation of the actin cytoskeleton and changes in the nuclear architecture, triggers a specific transcriptional program necessary for the metabolic adaptation of cells to the new environment. This response is mediated by modifications of the mitochondrial activity and dynamics and involves the crosstalk of mitochondria with other organelles as lysosomes and ER, where iron exchange takes place [72,74,79,112], also envisioning a possible role of iron in this signalling.

## 4. Iron Overload Causes Beta-Cell Dysfunction

**Figure 3:**
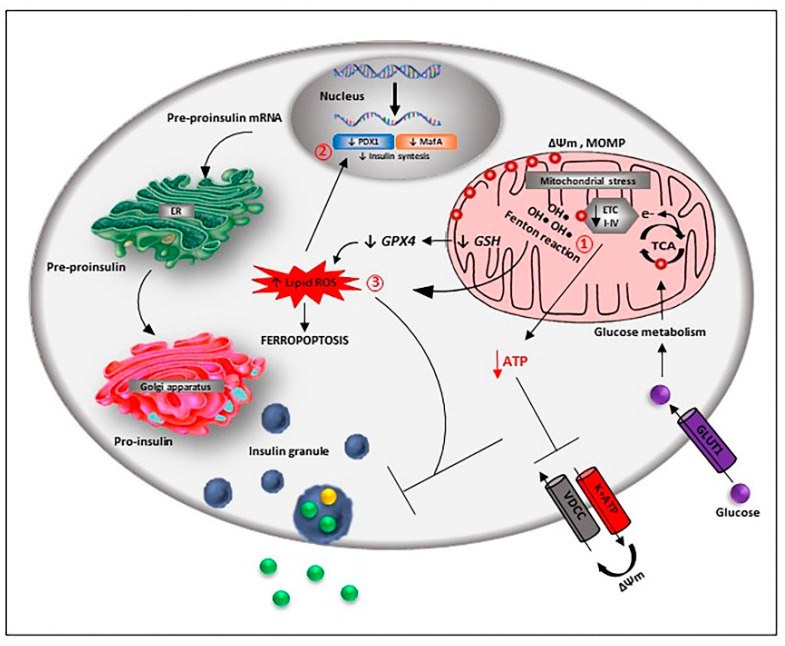
Iron-mediated toxic effects in beta-cells. Iron-mediated beta-cell toxicity is mainly due to reactive oxygen species (ROS) accumulation through Fenton’s reaction. ROS excess determines DNA, lipid and protein oxidation that causes mitochondrial damage, leading to insulin release and apoptosis (1). ROS also influence the activity of the pancreatic and duodenal homeobox 1 (PDX1) and V-Maf avian musculoaponeurotic fibrosarcoma oncogene homolog A (MafA), critical transcription factors for the control of insulin gene expression (2). Iron can induce beta-cell loss also through ferroptosis, a non-apoptotic cell death mechanism characterised by lipid ROS accumulation due to glutathione (GSH) depletion and consequent glutathione peroxidase-4 (GPX4) inhibition (3).

A normal iron level is required for proper beta-cell function, but its excess can be toxic, mainly through ROS formation and excessive activation of oxidative pathways (Figure 3). 

Hereditary hemochromatosis (HH) models have shown that iron accumulation affects more beta-cells than alpha- or delta-cells [113], probably because of the reduced levels of ROS-detoxifying enzymes in this cell subtype. 

Several mechanisms have been proposed to explain iron toxicity; a direct consequence of iron intracellular overload is that, as a positively charged ion, its entrance in mitochondria can depolarise the organelle membrane potential affecting both the electron transport chain and the energy supply for the insulin release [114,115]. Iron accumulation can generate ROS directly or indirectly. The redox active iron form (Fe^2+^) oxidises lipids in a Fenton’s-like reaction, resulting in a large amount of ROS that causes further ROS-mediated DNA and protein oxidation, decreased insulin synthesis and secretion, and apoptosis, as observed in the homeostatic iron regulator (*Hfe)* knock-out mouse model of HH [116]. It is noteworthy that other more severe iron overload models affecting hepcidin expression, such as the *Hamps* and the *Hjv* knock-out and the hepcidin-resistant model bearing the p.C326S mutation in ferroportin, although presenting pancreatic iron accumulation, do not show liver disease or endocrine problems, probably due to greater resistance to oxidative stress injury of mouse models [117].

The pancreatic and duodenal homeobox 1 (PDX1) and V-Maf avian musculoaponeurotic fibrosarcoma oncogene homolog A (MafA), two critical transcription factors involved in the control of insulin gene expression, are both targets for ROS [118], and decreased hepcidin expression in MIN6 cells leads to inhibited insulin synthesis via iron overload and decreased PDX1 expression [119,120].

Oxidant defences of beta-cells may also be reduced by iron overload through the inhibition of ROS-detoxification enzymes, such as the Mn^2+^ uptake and the Mn^2+^-dependent SOD activity [121]. 

Another mechanism by which iron overload may affect beta-cell function and survival is via amylin. Misfolding and aggregate deposition of hIAPP in the extracellular matrix and within beta-cells have been detected post-mortem in the pancreas of 90% of subjects affected by T2DM [122,123] where the polypeptide shows cytotoxic activity caused by the disruption of the cell membrane, perturbed ion homeostasis, endoplasmic reticulum stress, mitochondrial damage and dysfunction and final oxidative stress (see [124] as review). Intriguingly, iron has been shown to enhance amylin ß-sheet formation, triggering aggregate deposition [125]. Furthermore, as heme, it can bind to amylin, forming a complex that can lead to H_2_O_2_ formation via oxidative stress [126,127], thus fostering ROS-mediated beta-cell failure.

Iron may also contribute to beta-cell dysfunction and death through ferroptosis, a non-apoptotic form of cell death induced by the ion accumulation. Ferroptosis has been observed for the first time in cancer cells treated with the glutamate/cystine exchanger (X_c_) inhibitor erastin [128,129]. It is characterised by lipid ROS accumulation due to the glutathione peroxidase-4 (GPX4) inhibition caused by glutathione (GSH) depletion. In mouse islets, glucolipotoxic conditions have been reported to increase beta-cell iron import and cytosolic ROS formation [130]. Furthermore, pharmacological inhibition of GPX4 synthesis has been shown to induce glucose-mediated beta-cell dysfunction in vitro [131], while ferroptosis-inducing agents have been reported to compromise in vitro human islet viability and function [132], and the antidiabetic quercetin has been recently shown to reduce ferroptotic damages in pancreatic beta-cells of T2DM mouse models [133]. For the first time, our group was able to provide evidence that high extracellular levels of glutamate may represent an insult for beta-cells. Intriguingly, the glutamate action was not mediated by the excessive activation of ionotropic receptors, but rather by the glutamate-induced oxidative stress associated with alteration in the glutamate/cystine exchanger activity, GSH depletion and increased lipid peroxidation, a mechanism similar to ferroptosis [134,135]. 

Frataxin has also recently been implicated as a regulator of ferroptosis. In human fibrosarcoma HT-1080 cells [136], suppression of frataxin expression accelerates erastin-induced cell death, enhancing iron accumulation, lipid peroxidation and mitochondrial damage, events that were reverted by frataxin overexpression or pharmacological inhibition of ferroptosis. Accordingly, evidence of activation of a ferroptotic pathway of cell death has also been obtained in FDRA models, such as primary patient-derived fibroblasts, murine fibroblasts with FRDA-associated mutations and frataxin knockin/knockout murine fibroblasts [137]. 

In recent years, it has been also evidenced that beta-cell failure in T2DM can be related to cell de-differentiation processes [138,139]. Epigenetic modifications or changes in the transcription factor activity, and/or related variation in RNA or protein levels may determine loss of beta-cell gene expression or up-regulation of genes not normally expressed in mature beta-cells, like those expressed in islet progenitors or other mature islet cell types [139]. Interestingly, the Jumonji C-domain-containing histone demethylases, an epigenetic regulatory enzyme, is iron-dependent [140], and in hepatocarcinoma (HCC) specimens, a switch expression from TfR2 to TfR1 and overexpression of TfR1 have been associated with tumour dedifferentiation and poor prognosis [141], raising the interesting possibility that dedifferentiation may also be linked to alterations of iron metabolism.

## 5. Iron Dyshomeostasis Is Implicated in Diabetes

Evidence points to a direct link between dysregulation of iron metabolism and diabetic conditions. A previous section of this review has highlighted that increased ferritin levels can be detected in T2DM subjects [19] and in subjects affected by metabolic syndrome [142]. Increased incidence of diabetes (ranging from 20 to 60%) can be observed in patients with primary or secondary iron overload due to hereditary hemochromatosis or thalassemia because of both beta cell dysfunction and insulin resistance [143]. Moreover, subjects affected by aceruloplasminemia, an autosomal recessive disorder characterised by the lack of ceruloplasmin ferroxidase production with brain and liver accumulation of iron, also show increased risk of diabetes [144] as well as individuals affected by FRDA, the neurodegenerative disorder caused by deficiency of the mitochondrial iron chaperone frataxin [66].

Even a mild degree of iron excess, below levels typical of haemochromatosis or other iron-storage disorders, has been associated with an increased risk of gestational diabetes [145,146,147,148] and to an increased risk of developing non-alcoholic fatty liver disease (NAFLD) in the presence of metabolic syndrome [149].

Obesity, metabolic stress and T2DM are characterised by altered iron homeostasis: leptin-deficient ob/ob mice, that develop obesity and T2DM, show increased iron absorption and retention [150]. Hyperglycaemia has been reported to increase DMT1 expression and intestinal iron uptake in streptozotocin-induced diabetic mice, and increased brush-border DMT1 localisation has been observed in human diabetic intestinal biopsies [151]. Shu et al. have also reported a glucotoxicity-induced decrease in hepcidin expression, causing beta-cell failure by upregulation of TfR1 and DMT1 and consequent iron overload [120]. The iron chaperoning frataxin is reduced in islets from T2DM donors and in the humanised model of frataxin deficiency FDRA YG8R, causing iron overload in the mitochondria and beta-cell dysfunction [65].

In line with a causative role for iron in diabetic disease, dietary iron restriction has been reported to improve beta-cell function and glucose tolerance in ob/ob mice (84,144), and phlebotomy has been shown to improve insulin sensitivity, insulin secretion and glucose regulation in type 2 diabetes mellitus (T2DM) [152,153] even if clinical data, collected so far on iron depletion strategy, still appear inconclusive [154,155]. 

In summary, beta-cells need iron for their proper function. For that reason, they are equipped with a number of proteins involved in ion handling, such as the iron importers DMT1 and TfR1 and the iron storage protein ferritin. For a reason not yet fully understood, the level of these proteins changes during hyperglycaemic conditions, obesity and T2DM, thus resulting in iron accumulation which, through ROS production, causes impaired insulin synthesis, secretion and apoptosis, contributing to T2DM development and progression. Interestingly, some drugs proposed for the treatment of diabetes show a clear effect on iron homeostasis in beta-cells. For example, in the KIKO mouse model of FRDA, the incretin-mimetic exenatide has recently been confirmed to improve glucose homeostasis by increasing insulin release and by reducing oxidative stress through the induction of frataxin and Fe-S cluster protein expression [156]. Similarly, the antidiabetic thiazolidinediones prevents mitochondrial iron accumulation [157,158], further outlining the relationship between the control of iron homeostasis and beta-cell function preservation. 

## 6. Conclusions

Oxidative stress is one of the most important factors involved in diabetes pathogenesis, affecting pancreatic beta-cell function and survival, and iron, as a catalyser of ROS production by Fenton’s reaction, can represent one of the mediators of such a process. The relationship between iron dysregulation and beta-cell failure is established, and defects in iron storing and chaperon proteins have been associated with diabetic conditions. What needs to be clarified is whether beta-cell-specific sensitivity to iron overload is due to the low antioxidant capacity of these cells or if it is this incapacity that can lead to excessive accumulation of iron in hyperglycaemic and/or hyperlipidaemic conditions. Answers to these questions may help the definition of iron-control-based antidiabetic interventions aimed at the preservation of beta-cells.
